# The DI-SPME Method for Determination of Selected Narcotics and Their Metabolites, and Application to Bone Marrow and Whole Blood Analysis

**DOI:** 10.3390/molecules27134116

**Published:** 2022-06-27

**Authors:** Magdalena Świądro-Piętoń, Alicja Chromiec, Marcin Zawadzki, Renata Wietecha-Posłuszny

**Affiliations:** 1 Laboratory for Forensic Chemistry, Department of Analytical Chemistry, Faculty of Chemistry, Jagiellonian University, 2 Gronostajowa St., 30-387 Kraków, Poland; magda.swiadro@doctoral.uj.edu.pl (M.Ś.-P.); a.chromiec@doctoral.uj.edu.pl (A.C.); 2 Department of Forensic Medicine, Medical University in Wroclaw, 4 Jana Mikulicza-Radeckiego St., 50-345 Wrocław, Poland; marcin.zawadzki@umed.wroc.pl

**Keywords:** bone morrow, narcotics substances, SPME method, LC-MS technique

## Abstract

The present investigation utilised the quick and easy SPME/LC-MS method to determine selected narcotic substances and their metabolites in whole blood. The study included qualitative analysis and validation of the method. Analytes were determined in the linearity range of 25–300 ng/mL. The precision during and between days (in general CV < 13.41%), and the LOD which results in between 0.36 and 11.08 ng/mL, and the LOQ between 1.20 and 36.90 ng/mL were investigated. The validation results obtained, as well as the results of subsequent in-laboratory tests, confirmed the applicability of the method in the analysis of blood samples. An attempt to apply the method to the analysis of bone marrow samples has yielded promising results; however, more detailed studies are needed in this area.

## 1. Introduction

Over the past few decades, there has been an increase in the popularity and availability of stimulants. Despite the increasing variety of psychoactive substances, in recent years there has been a noticeable return to so-called classic drugs, such as cocaine and amphetamines. According to the data presented in the World Drug Report 2021 [1], a record for cocaine production was achieved in 2019. In total, 1.784 tons of the substance were produced (expressed at 100% purity), which is twice as much as in 2014. This is reflected in statistics on drug-related crimes. The 2021 European Drugs Report [2] shows a continuous increase in the number of crimes related to the possession or use of cocaine since 2016 (76,000 cases of this type were reported in 2019). In 2019, there was a decrease in the number of such crimes related to amphetamines (almost 55,000 cases), however, since 2009, there has been a general upward trend. In addition, the increasing number of deaths related to cocaine and amphetamines is alarming. For example, cocaine, in combination with opioids, caused more than half of the drug-related deaths reported in Spain in 2019; therefore, the development of methods for determining prohibited substances in biological samples allows a more accurate determination of the circumstances of the crime and verification of the testimony of the witnesses.

The success of the analytical process is highly dependent on the sample preparation step. Many procedures involve the separation of analytes from the matrix. The most commonly used methods include liquid–liquid extraction and solid-phase extraction [3]. However, the development of methods that meet the principles of Green Analytical Chemistry (GAC) collected by Galuszko et al. [4] is being pursued today. Solid-phase microextraction is an example of an extraction method that fits the GAC principles. The selection of appropriate parameters allows the simultaneous extraction of various compounds. This method makes it possible to reduce or, in some cases, completely exclude the need to use solvents; in addition, SPME fibers can be reused several times if appropriate cleaning procedures are implemented. It also does not require the use of toxic reagents and is safe for the analyst [3]. Nowak et al. [5] compared selected methods for the determination of tricyclic antidepressants in body fluids using the RGB12 algorithm. It accounts not only the GAC rules but also the area of application of the method, the important validation parameters, costs, simplicity of the procedure, and the time needed to perform the analysis. In this comparison, the DI-SPME-HPLC-MS method was the best, obtaining 96.3% of the points.

The SPME is based on the selective sorption of the determined compound on a thin layer of stationary phase that covers the fiber and then on its desorption. This is a non-exhaustive extraction method: the sorption process is carried out until the equilibrium concentration of the analyte between the sample and the stationary phase is established. The ratio of the concentrations in the two phases is independent of the amount of analyte in the sample so that quantitative analysis can be performed [6]. Solid-phase microextraction was initially used for samples with simple matrices. The appearance of new variants of the method after Direct Immersion SPME (Headspace SPME [7] and Membrane SPME [8])—as well as new types of sorbents—made it possible to analyze more complex samples, including environmental, food and biological samples [9]. The possibility of in vivo sampling opens the possibility of using DI-SPME for intraoperative research [10].

Attempts are also being made to automate the SPME process. Examples include in-tube SPME and fiber-in-tube SPME techniques. Both of them are based on some modifications of the extraction equipment concerning the location of sorbents. In the case of the former, the sorbent is placed on the inner wall of the capillary [11]. In the second, as the name suggests, a large number of very thin fibers that make up the sorbent are placed inside the capillary [12]. Modified devices give the possibility of creating a system that combines parts for the extraction and analysis of desorbed substances, which is a great automation of the process. However, a major limitation of the above-mentioned versions of the SPME technique is that, in most cases, they do not offer the possibility of analyzing samples with such a complex matrix as whole blood or bone marrow, as this would result in capillary clogging. Additional steps, such as deproteinization (which involves the use of additional amounts of reagents), and centrifugation are, therefore, required.

A large amount of information on the presence and stability of various substances in blood, as well as the possibility of correlation of the obtained results with pharmacological effects, make blood the commonly used biological material for toxicological analyses. Despite the fact that blood samples can be taken both from living and deceased subjects, various processes occurring in the human body after death or lack of the ability to collect blood samples require the use of other samples such as urine, vitreous humor, or bone marrow [13,14]. The possibility of determining xenobiotics in bone marrow has already been described by, for example, Snamina et al. [15] and de Souza Santos et al. [16]. This biological material can also be used in genetic analyses, to confirm death by drowning (diatom test) or to determine the time of death [17]. Bone marrow has several advantages compared to other materials. As a result of its rich blood supply and high lipid content, it can act as a “storehouse” for xenobiotics (especially lipophilic) introduced into the body. Its placement protects it from contamination as the bone remains intact. Furthermore, in the case of corpses in an advanced stage of decomposition or skeletonization, it is often one of the few materials available, due to its protection against the influence of fungi and bacteria, which delays the decay process [18,19,20].

The aim of this study was to present the application of the DI-SPME/LC-TOF-MS method for the detection and quantification of cocaine, amphetamine, mephedrone, and their metabolites in blood and bone marrow. The validation of the method and the intra-laboratory test were performed. The method was also used for the analysis of forensic case samples.

The compounds mentioned above were chosen because of their high popularity, as well as their similarity in the mechanism of action and symptoms of use. The use of DI-SPME for the extraction of cocaine, amphetamine, and their metabolites has already been described. The first attempts were made in the 1990s, but they involved the determination of one or two analytes simultaneously [21,22]. In the following years, methods for larger groups of analytes were developed. Usually, they involved the extraction of cocaine with its metabolites [23,24,25] or substances from the amphetamines group [26,27,28]. It is difficult to find an example of the application of solid phase microextraction to the group of analytes that contain both cocaine, amphetamine, and their metabolites. The biological material analyzed was usually hair, urine, or plasma, which provides information other than total blood analysis or requires additional sample preparation, and thus the use of additional amounts of reagents. Moreover, we were unable to find any example of an application of this method to the mephedrone.

## 2. Results and Discussion

### 2.1. Validation Parameters

**Figure 1:**
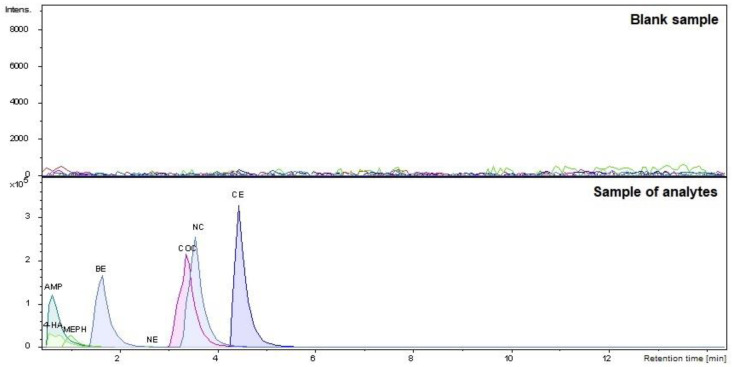
The chromatograms were obtained by applying the DI-SPME/LC-MS method for a blank blood sample and analyzed narcotic drugs at a concentration of 500 ng/mL. Peak assignment: 4-hydroxyamphetamine 4-HA (t_R_ = 0.56 min); amphetamine—AMP (t_R_ = 1.00 min); mephedrone—MEPH (t_R_ = 1.13 min); benzoylecgonine—BE (t_R_ = 1.64 min); norephedrine—NE (t_R_ = 2.59 min); cocaine—COC (t_R_ = 3.36 min); norcocaine—NC (t_R_ = 3.55 min); and cocaethylene—CE (t_R_ = 4.44 min).

The proposed DI-SPME/LC-TOF-MS methodology makes it possible to isolate and identify all analyzed narcotics. The results obtained from the determined analyzed drugs are presented in Figure 1.

To confirm the applicability of the DI-SPME/LC-MS method described in toxicological analyses, a validation process was carried out. Parameters such as linearity, the limit of detection (LOD), the limit of quantification (LOQ), intra- and inter-day precision, and carryover were investigated for all determined narcotics.

To determine the linearity of the method, a series of whole blood samples spiked with analytes at six concentration levels (25, 50, 100, 150, 200, 300 ng/mL) were prepared. An internal standard mix of two deuterated compounds was used at a concentration of 150 ng/mL. All analytes met the criterium of R^2^ > 0.99 and the linear models were obtained for all. The calculated LOD is in the range of 0.36–11.08 ng/mL. In the case of LOQ, the results oscillate between 1.20–36.90 ng/mL. Each value obtained indicates the possibility of determining 4-HA, AMP, MEPH, BE, NE, COC, NC, and CE at the minimum level needed to detect them.

The coefficient of variance values was calculated for all analytes at three concentration levels: 50, 150, and 300 ng/mL. As mentioned above, CV was used to express intra- and inter-day precision. In all cases, the CV values obtained met the validation protocol criteria (SWGTOX) [28]. In general, precision values, both intra-day and inter-day, did not exceed 13.41%. The only exception was NE, although it was at the lowest concentration level, for which the criterium is CV < 20%. This suggests that the proposed extraction method, such as DI-SPME, could be used for narcotics only in the case of qualitative and quantitative analysis in whole blood.

The carryover effect was also checked. In the case of BE, NC, 4-HA, and MEPH, no peaks were observed in the blank sample chromatogram examined right after the spiked sample at the concentration level of 300 ng/mL. For COC, CE, AMP, and NE, peaks were observed and compared with peaks obtained for spiked sample at the lowest concentration level examined (25 ng/mL). Area ratios were calculated. The highest S_0_/S_25_ ratio was obtained in the case of NE (2.54%), so the values for all narcotics met the protocol criteria and did not exceed 10%. Therefore, it can be concluded that the carryover in the analyzed samples of narcotics in the matrix was not observed. The parameters obtained are summarized in Table 1.

### 2.2. Intra-Laboratory Test

**Figure 2:**
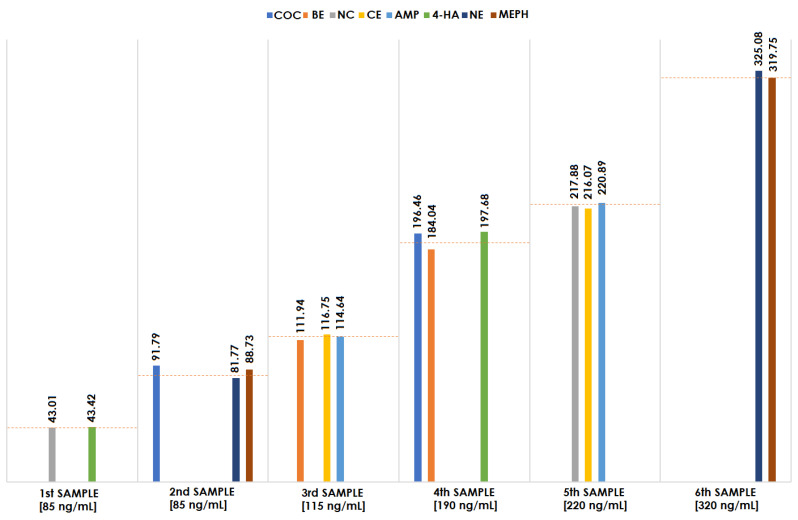
Results of the intra-laboratory test for blood samples and their comparison between the obtained and expected concentrations of the tested narcotics.

As mentioned, an intra-laboratory test was performed to verify the accuracy of the proposed methodology and it was divided into two stages. First, the other analyst prepared only spike blood samples at different concentrations: first sample–42 ng/mL; second sample–85 ng/mL; third sample–115 ng/mL; fourth sample–190 ng/mL; fifth sample–220 ng/mL; and the sixth sample–320 ng/mL. In the next step, each sample has been tested according to the scheme described in the section. The results obtained during the analysis are presented in Figure 2.

A comparison of the concentrations determined, and the concentrations declared by the person who prepared the examined samples (marked with red lines in Figure 2) confirms the effectiveness of the method. It should be noted that the concentration of analytes in sample number 6 was outside the linear range tested by the method. However, the test results for this sample were also similar to the declared concentration, indicating the possibility of determining analytes also in higher concentrations. It may be concluded that the DI-SPME method is a good technique for isolating narcotics from blood, even samples of unknown concentrations.

**Figure 3:**
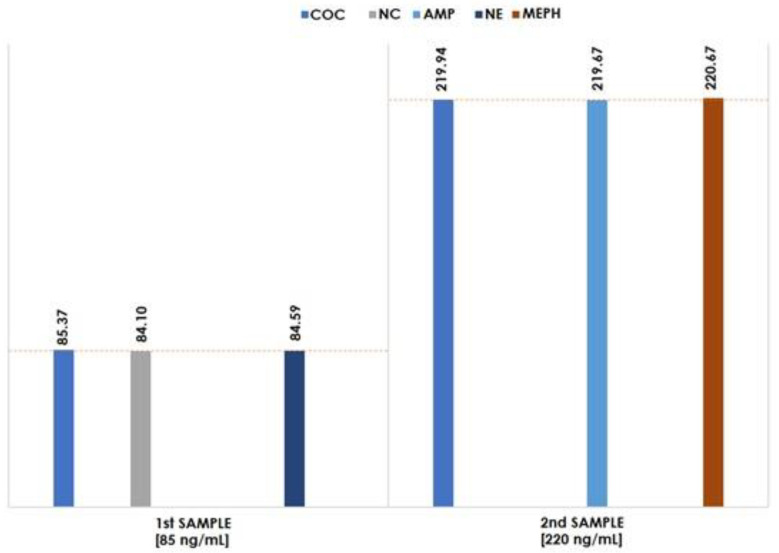
Results of intra-laboratory test for bone marrow samples and their comparison between the obtained and expected concentration of the narcotics tested.

The second part of the conducted intra-laboratory test was to examine bone marrow aspirate samples, both spiked with three chosen narcotics, of course, in different concentrations: first sample–85 ng/mL; and second sample–220 ng/mL. The results are shown in Figure 3.

When comparing the concentrations determined to the concentrations declared by the person who prepared the examined samples (marked with red lines in Figure 3), it can be concluded that they are close to each other. The developed method can be used for different matrices, not only for blood but also for bone marrow analysis. In the long term, research on that matter should be extended to the analysis of more bone marrow samples containing all narcotics at different concentration levels.

### 2.3. Post-Mortem Case Sample

**Figure 4:**
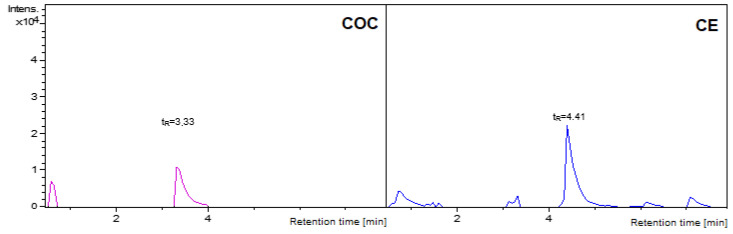
Chromatogram confirming the detection of cocaine (COC) and cocaethylene (CE) in the postmortem case sample tested.

The validated DI-SPME/LC-MS method was used for the analysis of a real sample collected post-mortem, which was not analyzed by a different technique. During the qualitative analysis based on detected mass and retention time, two of the narcotics of interest were identified—cocaine. (COC t_R_ = 3.36 min) and cocaethylene (CE t_R_ = 4.44 min)—see Figure 4.

In addition to qualitative analysis, a quantitative analysis was also performed. The final concentration of COC was determined at 132.46 and CE at 97.94 ng/mL. Therefore, the results obtained from the case sample confirm the effectiveness of the proposed method for the qualitative analysis of samples of completely unknown composition; furthermore, it enables the determination of the exact concentration of the detected narcotic drugs. However, in this case, this sample should be analyzed using other methods to verify the concentration obtained of cocaine and its cocaethylene metabolite.

## 3. Materials and Methods

### 3.1. Chemicals and Reagents

Standard solutions of analytes: cocaine (COC), norcocaine (NC), and cocaethylene (CE) in acetonitrile, amphetamine (AMP), 4-hydroxyamphetamine (4-HA), norephedrine (NE), benzoylecgonine (BE), and mephedrone (MEPH) in methanol were purchased from Lipomed AG (Arlesheim, Switzerland). Standard solutions of internal standards: 4-hydroxyamphetamine-d5 (4-HA-d5) and fluoxetine-d6 (FLUOX-d6) in methanol were also purchased from Lipomed AG (Arlesheim, Switzerland).

LC-MS grade solvents: acetonitrile, methanol, and isopropyl alcohol were obtained from Honeywell–Riedel-de Han (Morristown, NJ, USA), sodium hydroxide was obtained from Fluka Analytical (Seelze, Germany) and formic acid was obtained from Merck (Darmstadt, Germany). Ultrapure deionized water (18.2MΩ⋅cm, TOC < 5 ppb) was obtained using the Mili-Q Plus system (Milipore, Bedford, MA, USA).

During this study, the following equipment and devices were used: SPME-LC Probe 45 μm C18-Silica fibers (Supelco) from Merck (Darmstadt, Germany); HPLC vials (1.5 mL) and inserts (200 μL) from VWR (Randor, PA, USA), dust-free material (Kimberly-Clark Corporation; Irving, TX, USA), manual pipettes from Sartorius AG (Göttingen, Germany), Digital Vortex Mixer and Thermal Shake Touch from VWR (Randor, PA, USA), Concentrator plus from Eppendorf AG (Hamburg, Germany), Ultimate 3000 RS liquid chromatography system (UHPLC; Dionex, Sunnyvale, CA, USA) equipped with a Hypersil Gold Phenyl column (50 mm × 2.1 mm ID, particles 1.9 μm) from Thermo Scientific (Bruker, Bremen, Germany) coupled to a mass spectrometer (MS) equipped with electrospray ionization source (ESI) and MicroTOF-Q II time

The following software was used during the research: Chromeleon 6.8 (Dionex), HyStar 3.2, MicrTOFcontrol and Compass DataAnalysis software (Bruker Bremen, Germany), and IsotopePattern software (Bruker, Bremen, Germany).

### 3.2. Sample Collection

The biological material used during the optimized research was drug-free human blood provided by the Kraków Blood Bank, Poland. Human bone marrow analysis was performed using biological material provided by the Department of Forensic Medicine of Wroclaw Medical University (in accordance with Bioethical Commission no. 1072.6120.303.2018).

Blood samples and bone marrow aspirates were prepared 24 h prior to analysis. In the case of preparing a calibration curve and determining validation parameters, blood samples were spiked with a known amount of drug solution and an internal standard solution, while the case sample was spiked only with an internal standard solution. All samples were stored in a freezer at a temperature of −20 °C until use.

### 3.3. Instrumentations

The operating parameters of liquid chromatography coupled with the mass spectrometer (LC-MS) are presented in Table 2.

The gradient elution program was started by gradually reducing the content of phase A (0.1% formic acid solution) in the eluent from 85 (0 min) to 60% (4 min) and simultaneously increasing the content of phase B (acetonitrile) from 15 (0 min) up to 40% (4 min). This composition of the mobile phase was maintained until the 7th minute, after which the content of phase A was gradually reduced to 30% and the content of phase B was increased to 70% (from the 7th to the 10th minute). Then the proportion of phase A in the mixture increased to 85% and phase B was reduced to 15% (in the range of 10–12.5 min). This composition of eluent was kept until the end of the measurement.

### 3.4. DI-SPME Procedure

**Figure 5:**
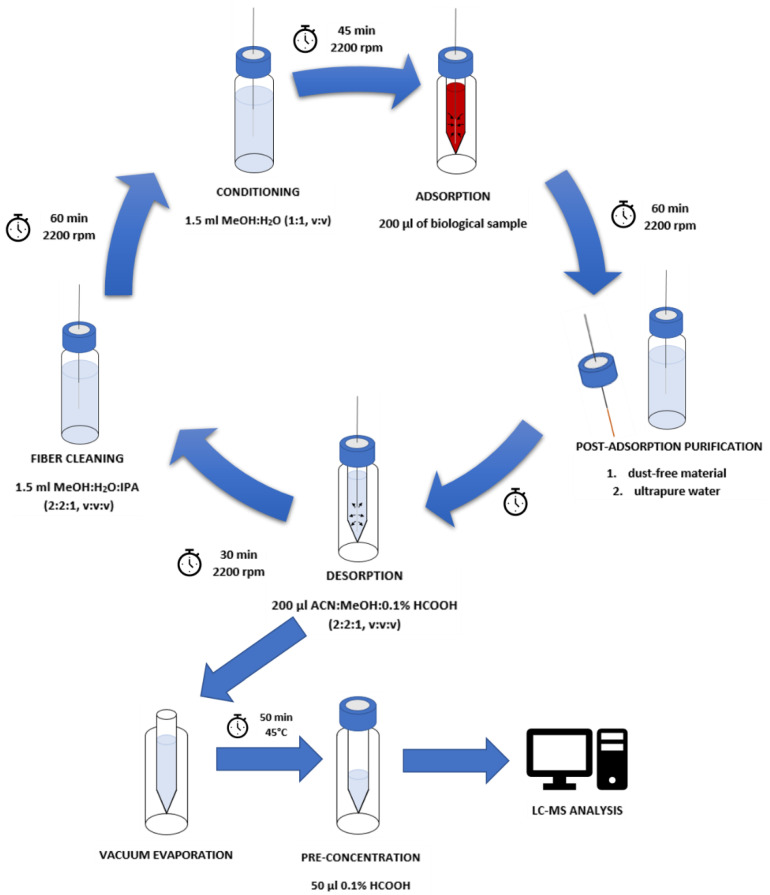
Procedure of the DI-SPME method of sample preparation and fiber cleaning.

In the proposed research, the methodology described by Majda et al. [29] has been modified, especially for the identification of narcotics in human blood. The first step of the extraction process was fiber conditioning. It was performed according to the manufacturer’s suggestion. The fibers were placed in vials containing 1.5 mL of conditioning solution (methanol: water, 1:1, *v*/*v*) and then the whole was shaken at 2200 rpm, at room temperature for 45 min. The fibers were then transferred to 1.5 mL vials with inserts containing 200 µL of biological material. Adsorption was carried out at room temperature for 60 min with a continuous shaking of 2200 rpm. The third step of the procedure was fiber purification. For this purpose, wiping the fibers with dust-free tissues and rinsing with ultrapure water with Vortex, agitation was introduced into the procedure. After that, the fiber was ready for the desorption step. It was carried out by placing fibers in 1.5 mL vials with inserts containing 200 µL of desorption solution (acetonitrile: methanol:0.1% formic acid solution, 2:2:1, *v*/*v*/*v*) for 30 min with 2200 rpm agitation. The fibers were then placed in vials filled with 1.5 mL of cleaning solution (methanol: water: isopropanol, 2:2:1, *v*/*v*/*v*) to prepare them for the next analysis. The desorption solutions were evaporated using a vacuum evaporator for 50 min at 45 °C. After that, 50 µL of the mobile phase (0.1% formic acid solution) was added to the inserts and mixed using a vortex shaker. The samples prepared in this way were analyzed with the use of the LC-TOF-MS system. The procedure described above is presented in Figure 5.

### 3.5. Validation Parameters

The validation procedure was carried out according to standard practices for the validation of the method by the Scientific Working Group on Forensic Toxicology (SWGTOX) [30]. Blood samples were used as a matrix. The following parameters were determined: linearity, the limit of detection (LOD), the limit of quantification (LOQ), intra-day and inter-day precision, and carryover.

The interpolative internal standard method was used for the calibration process. To create calibration curves, linear regression of ratios between the analyte peak area and the corresponding internal standard peak area (IA/IIS) was made. The linearity was checked within the range of 25–300 ng/mL. It was assumed that the coefficient of determination (R^2^) was not less than 0.995.

The limit of detection was the concentration of the analyte for which signal (S) to noise (N) ratio: S/N = 3, and the quantification limit was the concentration of analyte for which S/N = 10.

The precision of the method was tested at three concentration levels: 50, 150, and 300 ng/mL. The precision during the day was determined of 3 samples. The precision between days was also determined. It was calculated from a total of 12 samples analyzed over 4 days (three samples per day). It was expressed as the coefficient of variation (CV) and calculated using Equation (1)

(1) $$\mathrm{CV}=\frac{s}{x_{am}}\cdot 100\%$$

where: *s*—standard deviation; *x_am_*—arithmetic mean. According to the validation protocol [28], CV should not exceed 15%, but at the lowest concentration level, it should be less than 20%.

To evaluate the carryover effect, the blank sample was examined immediately after the sample at the highest concentration level (300 ng/mL). According to the regulations, the signal measured for this blank sample (S_0_) should not be higher than 10% of the average signal for the lowest concentration (S25), in that case: 25 ng/mL.

### 3.6. Intra-Laboratory Test

The developed method was tested by examining blood and bone marrow samples of unknown composition prepared by another analyst. Six blood samples with analytical concentrations in the range of 25–350 ng/mL were tested. Each of the samples contained two or three selected compounds. In this case of bone marrow, two samples of unknown composition, also prepared by another analyst, were analyzed. Each sample contained three selected analytes. The calculations were performed using the linear model obtained at the beginning of the investigation of blood samples.

### 3.7. Case Sample

The most interesting aspect of this investigation was the analysis of a case sample, provided by the Wroclaw Department of Forensic Medicine. It was an unknown composition sample that, according to the information obtained, could contain some of the narcotics analyzed. Therefore, for identification, the proposed DI-SPME/LC-MS methodology was applied.

## 4. Conclusions

The developed method, consisting of the DI-SPME method (sample preparation and extraction method) and liquid chromatography coupled with a mass spectrometer (sample analysis stage), meets the qualitative and quantitative requirements for the determination of some narcotic substances, such as cocaine, amphetamine, mephedrone, and their metabolites. Moreover, the applied SPME technique allows the amounts of organic solvents to be decreased in the extraction process. The biological material most applied to determinate narcotic substances was usually hair, urine, or plasma. The described research confirms the effectiveness of this method DI-SPME/LC-MS for the analysis of blood and bone marrow for the identification of cocaine, amphetamine, mephedrone, and their metabolites. An important advantage of this method, especially in the case of forensic samples, is also the small amount of biological material (200 µL) necessary to perform a single analysis. Due to this, the DI-SPME/LC-MS method is good alternative to those routinely employed in forensic examinations. In this publication, the results of the validation process, as well as the intra-laboratory test, showed enormous potential for identification of low concentrations from insignificant amounts of cases. The innovative aspect of this research concerns the analysis of the mephedrone. No example of an application of the SPME method to the identification of mephedrones is available. Thus, it may be concluded that the proposed methodology is one of the first in this regard.

## Figures and Tables

**Table 1:** Summary of the validation parameters for the DI-SPME/LC-MS method.

Parameter.	COC	BE	NC	CE	AMPH	4-HA	NE	MEPH
**linearity**	LOQ-300							
**a**	0.0041	0.0012	0.0097	0.0095	0.0025	0.0036	0.0037	0.0024
**b**	0.0467	0.1027	0.0365	0.1297	0.0275	0.0038	0.0848	0.1175
**R^2^**	0.9995	0.9958	0.9928	0.9969	0.9985	0.9967	0.9977	0.9905
**LOD [ng/mL]**	0.6	1.1	0.5	0.4	11.1	9.0	5.9	6.2
**LOQ [ng/mL]**	2.1	3.6	1.7	1.2	36.9	29.9	19.7	20.7
**Precision 50 [ng/mL], CV [%]**								
**interday**	6.2	7.7	5.8	6.4	7.1	7.3	7.7	11.1
**intraday**	5.2	7.	5.7	7.4	9.5	6.2	18.2	8.2
**Precision 150 [ng/mL], CV [%]**								
**interday**	2.5	6.2	3.5	5.5	8.5	3.2	3.9	4.5
**intraday**	4.8	4.8	3.0	4.7	11.1	3.9	13.4	7.5
**Precision 300 [ng/mL], CV [%]**								
**interday**	3.6	2.5	0.8	4.7	6.8	1.2	5.5	1.9
**intraday**	9.1	2.2	6.6	1.1	11.3	5.4	11.0	4.8
**carryover, S_0_/S_25_ [%]**	1.1	0 *	0 *	0.7	0.6	0 *	2.54	0 *

* No peaks.

**Table 2:** LC-MS parameters.

LC-MS Parameters	Value
column temperature;	35 °C
mobile phase	phase A: 0.1% formic acid solution phase B: acetonitrile
injection volume	5 µL
flow rate	0.3 mL/min
time of procedure	17 min.
ionization mode;	positive
capillary voltage	4.5 kV
nebulizer pressure	2.5 bar
dry gas temperature	200 °C
dry gas flow	5.5 L/min
mass range	50–800

## Data Availability

Data sharing not applicable.

## Sample Availability

Samples of the compounds are not available from the authors.
